# Vertical Alignment of Liquid Crystal on Sustainable 2,4-Di-*tert*-butylphenoxymethyl-Substituted Polystyrene Films

**DOI:** 10.3390/polym14071302

**Published:** 2022-03-23

**Authors:** DaEun Yang, Chowon Jin, Hyo Kang

**Affiliations:** BK-21 Four Graduate Program, Department of Chemical Engineering, Dong-A University, 37 Nakdong-Daero 550 Beon-gil, Saha-gu, Busan 49315, Korea; 1830133@donga.ac.kr (D.Y.); a3329798@naver.com (C.J.)

**Keywords:** liquid crystal, polystyrene, orientation layer, vertical, 2,4-di-*tert*-butylphenol

## Abstract

We synthesized sustainable 2,4-di-*tert*-butylphenoxymethyl-substituted polystyrenes (PD*t*BP#, # = 88, 68, 35, and 19, where # is molar percent contents of 2,4-di-*tert*-butylphenoxymethyl moiety), using post-polymerization modification reactions in order to study their liquid crystal (LC) alignment behaviors. In general, LC cells fabricated using polymer film with higher molar content of 2,4-di-*tert*-butylphenoxymethyl side groups showed vertical LC alignment behavior. LC alignment behavior in LC cell was related to the surface energy of the polymer alignment layer. For example, when the total surface energy value of the polymer layer was smaller than about 29.4 mJ/m^2^, vertical alignment behaviors were observed, generated by the nonpolar 2,4-di-*tert*-butylphenoxymethyl moiety with long and bulky carbon groups. Orientation stability was observed at 200 °C in the LC cells fabricated using PD*t*BP88 as the LC alignment layer. Therefore, as a natural compound modified polymer, PD*t*BP# can be used as a candidate LC alignment layer for environmentally friendly applications.

## 1. Introduction

Liquid crystals (LCs) are mesophase materials between the crystalline and the liquid states and have an anisotropic shape [1]. LC molecules also exhibit excellent physicochemical properties depending on the molecular positional and/or orientational order [2,3,4,5]. LCs are broadly classified into two types, lyotropic and thermotropic LC, according to their physical parameters and the environment in which they exhibit liquid crystal properties [6,7,8,9,10]. Lyotropic LCs (or lyomesophases) consisting of amphiphiles have a tendency to form self-assembled structures depending on their concentration in solution. The physicochemical properties of lyotropic LCs and their intrinsic structures, including lamellar, hexagonal, and cubic phases, make them suitable as drug delivery agents in pharmaceutical sciences in a wide range of applications [11,12,13,14,15,16]. In particular, the hexagonal and cubic phases are of high interest in the field of drug delivery agents owing to their ability to control or maintain the release of hydrophilic and hydrophobic drug molecules with a wide range of molecular weights [17,18,19]. Thus, by controlling the concentration of the lyotropic LC system, active ingredients can be uploaded to pharmaceuticals with different polarities and sizes. Thermotropic LCs exhibit various liquid crystalline phases, which are formed by change in temperature. Thermotropic LCs consist of rod-shaped molecules and are subdivided into three main classes: nematic, smectic, and cholesteric. Among them, nematic LCs are the simplest liquid crystal phase and have a characteristic long-range orientational order expressed by the unit vector *n* [20]. Nematic LCs are widely applied in optical devices such as adaptive lenses, spatial light modulators, and polarization gratings [21]. Nematic LCs are typically sandwiched between two glass substrates whose surfaces are covered with an alignment layer. The orientation surface controls the nematic LC orientation via an external electric and/or magnetic field in the LC cell.

According to the degree of difference of the LC indicator of the cell, the orientation of LC molecules is classified into two types: one-directional (planar, tilted, and vertical) and multi-directional (hybrid, bend, splay, and twist). Orientation of LC molecules in one direction can play an essential role in industrial applications and scientific research [22,23]. The most well-known example of this effort is the development of liquid crystal displays (LCDs) that include an alignment layer, which is generally used in portable electronic devices such as electronic toys, digital cameras, and smartphones. Typical display panel technologies such as twisted nematic mode, which are commonly used in LCDs, have disadvantages in that the viewing angle is narrow, and the contrast ratio is low [24,25]. Therefore, other modes have been developed, including in-plane switching (IPS), mode/fringe-field switching (FFS), and mode/vertical alignment (VA) mode that exhibits wide viewing angles and high contrast ratios [26,27,28,29,30]. Among these modes, the VA mode has a particularly high contrast ratio due to the minimum delay [31]. The vertical alignment of LC molecules can be explained using the correlation between the surface energy of the alignment layer (γ_s_) and the surface tension of the liquid crystal (γ_lc_) according to the semiempirical Friedel–Creagh–Kmetz rule [32,33,34]. Therefore, when γ_lc_ > γ_s_, liquid crystal materials can be oriented vertically to the substrate surface. Polyimide (PI), which has excellent thermal and chemical stability, has a side chain such as a long alkyl group and has been used to implement vertical alignment of LC molecules [35,36,37]. However, it is difficult to produce flexible plastic products because the post-baking and washing temperatures required for preparing the alignment layer from the PI derivative are too high [38,39]. Recently, a vertically aligned LC layer using a polystyrene (PS) derivative synthesized through a polymer substitution reaction has attracted much attention in the electro-optical field including flexible displays due to its advantages of excellent optical transparency and low-temperature processability [40,41]. PS derivatives, grafted with long alkyl groups, natural extracts, etc., have been studied to orient LC molecules vertically to the substrates using a non-contact method [42,43]. For example, vertical orientation of LC molecules has been shown in previous studies in LC cells prepared with PS derivatives substituted with long alkyl groups, natural extracts such as eugenol, vanillin, and isoeugenol [44,45,46]. This is due to the long alkyl group of the natural extract, which is related to the low surface energy as a result of the steric effect of the alkyl group on the surface of the polymer film [47].

The synthesis of bulky alkylated phenols for a wide range of applications is an important research area in a variety of fields [48]. One of the bulky alkylated phenols, 2,4-di-*tert*-butylphenol belongs to a class of phenolic compounds with two *tert*-butyl substituents at positions 2 (*ortho*) and 4 (*para*). As a common natural product, 2,4-di-*tert*-butylphenol is found in essential oils, and exhibits high toxicity to organisms such as bacteria, fungi, etc. [49,50]. It has been broadly used in acaricide, herbicide, cancer drugs, and food additives [51,52]. Because 2,4-di-*tert*-butylphenol has a phenolic component, it has been reported to contain various biological and chemical activities [53,54,55]. For example, 2,4-di-*tert*-butylphenol has biological activity and can control bacteria that play a significant role in biocorrosion and biofilm, which causes enormous economic losses in industrial environments [56,57,58]. In addition, as one of the main chemical activities of 2,4-di-*tert*-butylphenol, its antioxidant activity has received much attention in the medical, food, and pharmaceutical industries [59,60,61]. 2,4-Di-*tert*-butylphenol have been investigated to block oxidative stress caused by reactive oxygen species (ROS) and free radicals [62]. Therefore, the synthesis of this bulky 2,4-di-*tert*-butylphenol is of increasing interest in a variety of applications due to its diverse properties.

In this paper, we synthesized environmentally friendly 2,4-di-*tert*-butylphenoxymethyl-substituted polystyrene (PD*t*BP#) to obtain vertical alignment of LCs and investigated the effect of molar content of side groups on LC alignment behavior. The synthesis and characterization of these natural polymers and the optical properties of LC cells fabricated using these polymer films were also studied.

## 2. Experimental Section

### 2.1. Materials

4-Chloromethylstyrene (CMS), 2,4-di-*tert*-butylphenol, chloroform-d (CDCl_3_, used as a solvent in nuclear magnetic resonance, NMR), and potassium carbonate were obtained by Aldrich Chemical Co. (Seoul, Korea). 4′-Pentyl-4-biphenylcarbonitrile (5CB) (*n_e_* = 1.7360, *n_o_* = 1.5442, and *Δε* = 14.5, where *n_e_* is the extraordinary refractive index, *n_o_* is the ordinary refractive index, and *Δε* represent dielectric anisotropy), silica gel, and ethanol were purchased from Merck Co. (Seoul, Korea). *N,N’*-Dimethylacetamide (DMAc) were dried over 4 Å molecular sieves. The methanol was purchased by Daejung Chemicals & Metals Co. (Siheung, Korea). Tetrahydrofuran (THF) was refluxed over benzophenone and sodium and then distilled. 2,2′-Azobisisobutyronitrile (AIBN) was purchased from Daejung Chemicals & Metals Co. (Siheung, Korea) and was purified by crystallization from methanol. CMS was purified by silica gel column chromatography with hexane to remove inhibitors and impurities. All other solvents and reagents were purchased from commercial supplier sources and used as received.

### 2.2. Preparations of Poly(4-chloromethylstyrene) (PCMS)

Poly(4-chloromethylstyrene) (PCMS) was synthesized by general free radical polymerization of CMS (15 g, 0.1 mol) with AIBN (0.15 g, 1 mmol, 1 wt.% compared to CMS) under a nitrogen atmosphere at 60 °C for 24 h using a magnetic bar. The mixture in solution was cooled to room temperature and then poured into methanol (800 mL) to obtain a white precipitate. This precipitate was further purified by Soxhlet extraction using hot methanol overnight to remove the remaining monomer (CMS) and low molecular weight PCMS. The chemical composition of the obtained PCMS was confirmed using ^1^H NMR spectroscopy. In addition, the chemical composition of PCMS was confirmed in detail using ^13^C NMR spectroscopy in the Supplementary Materials.

^1^H NMR of PCMS (400 MHz, CDCl_3_, *δ*/ppm): *δ* = 1.2–1.8 (–*CH_2_*–*CH*–Ph–, 3H), 4.3–4.6 (–Ph–*CH_2_*–Cl, 2H), 6.1–7.2 (CH_2_–CH–*PhH*–CH_2_–, 4H).

### 2.3. Preparations of 2,4-Di-tert-butylphenoxymethyl-Modified Polystyrene (PDtBP#)

The series of 2,4-di-*tert*-butylphenoxymethyl-modified polystyrene (PD*t*BP#), where # represents the molar content (%) of 2,4-di-*tert*-butylphenoxymethyl moiety in the side group were studied. The synthesis of 2,4-di-*tert*-butylphenoxymethyl-modified polystyrene (PD*t*BP88) is considered a representative example. A mixture of 2,4-di-*tert*-butylphenol (0.36 g, 1.74 mmol, 150 mol% compared to with PCMS) and potassium carbonate (0.29 g, 2.09 mmol, 120 mol% compared to 2,4-di-*tert*-butylphenol, used as a substituent) was heated to 75 °C in DMAc (30 mL). A solution of PCMS (0.30 g, 1.97 mmol) and DMAc (20 mL) was added to this mixture, and the mixture was stirred under a nitrogen atmosphere at 70 °C for 24 h using a magnetic bar. The solution mixture was poured into methanol (200 mL) to obtain white precipitate. The white precipitate was further purified by reprecipitation several times in methanol and then washed using hot methanol to remove remaining salts and potassium carbonate. After vacuum drying, PD*t*BP88 was obtained in a yield of 80% or more. Other polystyrene derivatives containing 2,4-di-*tert*-butylphenoxymethyl side groups were synthesized using the same process used for the preparation of PD*t*BP88, except for differing amounts of 2,4-di-*tert*-butylphenol in the reaction. For example, PD*t*BP68, PD*t*BP35, and PD*t*BP19 were prepared with 0.28 g (1.36 mmol), 0.14 g (0.68 mmol), and 0.08 g (0.37 mmol) of 2,4-di-*tert*-butylphenol, respectively, using excess amounts of potassium carbonate (120 mol% relative to 2,4-di-*tert*-butylphenol). The chemical compositions of the obtained PD*t*BP#s were confirmed using ^1^H NMR spectroscopy. In addition, the chemical compositions of PD*t*BP#s were confirmed in detail using ^13^C NMR spectroscopy in the Supplementary Materials.

^1^H NMR of PD*t*BP88 (400 MHz, CDCl_3_, *δ*/ppm): *δ* = 1.2–1.3 (*–*C*(CH_3_)_3_–*, 18H), 1.3–1.6 (*–CH_2_–CH–*Ph*–*CH_2_*–*O*–*, 3H), 4.8–5.6 (*–*Ph*–CH_2_–*O*–*Ph*–*, 2H), 6.1–7.3 (*–*CH_2_*–*CH*–PhH–*CH_2_*–*O*–PhH–*, 7H).

^1^H NMR of PD*t*BP68 (400 MHz, CDCl_3_, *δ*/ppm): *δ* = 1.2–1.3 (*–*C*(CH_3_)_3_–*, 18H), 1.3–1.6 (*–CH_2_–CH–*Ph*–*CH_2_*–*O*–*, 3H), 4.8–5.1 (*–*Ph*–CH_2_–*O*–*Ph*–*, 2H), 6.1–7.3 (*–*CH_2_*–*CH*–PhH–*CH_2_*–*O*–PhH–*, 7H).

^1^H NMR of PD*t*BP35 (400 MHz, CDCl_3_, *δ*/ppm): *δ* = 1.2–1.3 (*–*C*(CH_3_)_3_–*, 18H), 1.3–1.6 (*–CH_2_–CH–*Ph*–*CH_2_*–*O*–*, 3H), 4.8–5.1 (*–*Ph*–CH_2_–*O*–*Ph*–*, 2H), 6.1–7.3 (*–*CH_2_*–*CH*–PhH–*CH_2_*–*O*–PhH–*, 7H).

^1^H NMR of PD*t*BP19 (400 MHz, CDCl_3_, *δ*/ppm): *δ* = 1.2–1.3 (*–*C*(CH_3_)_3_–*, 18H), 1.3–1.6 (*–CH_2_–CH–*Ph*–*CH_2_*–*O*–*, 3H), 4.8–5.1 (*–*Ph*–CH_2_–*O*–*Ph*–*, 2H), 6.1–7.3 (*–*CH_2_*–*CH*–PhH–*CH_2_*–*O*–PhH–*, 7H).

### 2.4. Film Preparation and LC Cell Assembly

Five solutions of PD*t*BP88, PD*t*BP68, PD*t*BP35, PD*t*BP19, and PCMS were prepared at 1 wt% in THF and filtered using a poly(tetrafluoroethylene) membrane having a pore size of 0.45 μm. The polymer thin film was prepared by spin-coating (spinning speed 2000 rpm, spin time 90 s) on a 2.0 × 2.5 cm^2^ glass substrate. LC cells were fabricated by assembling two glass slides coated with a polymer film using a 4.25 μm-thick spacer and filled with pure nematic LC (5CB). The fabricated LC cell was sealed with an epoxy glue.

### 2.5. Instrumentation

^1^H nuclear magnetic resonance (^1^H NMR) spectroscopy and ^13^C nuclear magnetic resonance (^13^C NMR) spectroscopy using an MR400 DD2 (Agilent Technologies, Inc., Santa Clara, CA, USA) were used to identify the synthesized structure. Fourier transform-infrared (FT-IR) spectrometry (NICOLET iS20, Thermo Fisher Scientific, Waltham, MA, USA) was used to identify the synthesized structure. Differential scanning calorimetry (DSC) using a Q-10 (TA Instruments, Inc., New Castle, DE, USA) was used to confirm the thermal properties of synthesized materials. The glass transition temperature (*T_g_*) was detected during the second heating run over the range −40 °C to 200 °C. Polarized optical microscopy (POM) images of LC cells using a Nikon Eclipse E600 POL (NIKON, Inc., Tokyo, Japan) with polarizer and Nikon Coolpix 995 digital camera (NIKON, Inc., Tokyo, Japan) were used to characterize the synthesized materials. The contact angle of methylene iodide, distilled water on the polymer film was measured at room temperature with KRÜSS DSA10 (KRÜSS Scientific Instruments Inc., Hamburg, Germany) contact angle analyzer connected with droplet analysis software. The average volume of the droplet used for measuring the contact angle was 5 μL, and after dropping the droplet, it was measured in an equilibrium region where the contact angle was constant. The contact angle for each sample was measured at least four times for three independently produced films and averaged. The surface energy value was calculated using the contact angle based on the Owens–Wendt’s Equation as

γ*_sl_*_=_ γ*_s_* + γ*_l_* − 2(γ*_s_^d^*γ*_l_^d^*)^1/2^ − 2(γ*_s_^p^*γ*_l_^p^*)^1/2^ (1)

where γ*_sl_* is the interfacial energy of the solid/liquid interface, γ*_s_* is the surface energy of the solid, γ*_l_* is the surface energy of the liquid, γ*_l_^d^* and γ*_l_^p^* are the known surface energy for the test liquids, γ*_s_^d^* and γ*_s_^p^* can be calculated from the measured static contact angles [63].

## 3. Results and Discussion

### 3.1. Synthesis and Characterization of 2,4-Di-tert-butylphenoxymethyl-Modified Polystyrene

**Figure 1:**
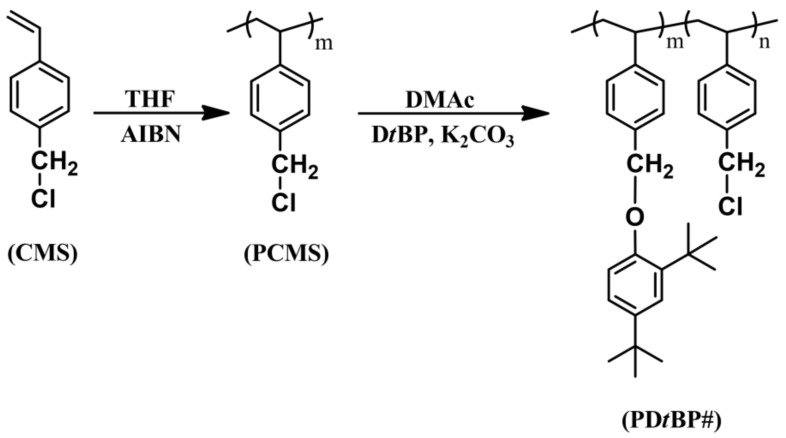
Synthetic route of 2,4-di-*tert*-butylphenoxymethyl modified polystyrene (PD*t*BP#), where # indicates the mole percent of 2,4-di-*tert*-butylphenoxymethyl containing monomeric units in the polymer.

**Figure 2:**
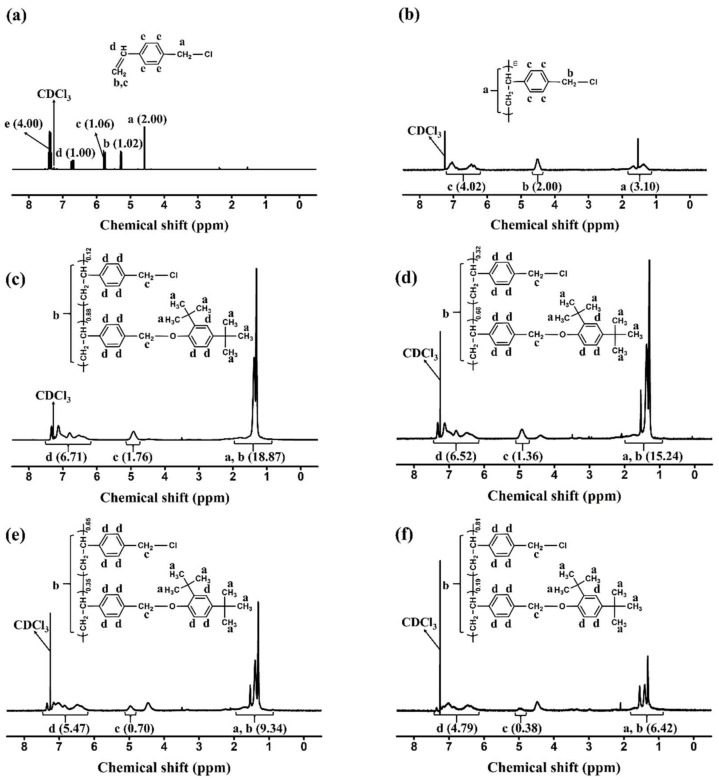
^1^H nuclear magnetic resonance (^1^H NMR) spectra of: (**a**) CMS, (**b**) PCMS, (**c**) PD*t*BP88, (**d**) PD*t*BP68, (**e**) PD*t*BP35, and (**f**) PD*t*BP19.

**Figure 3:**
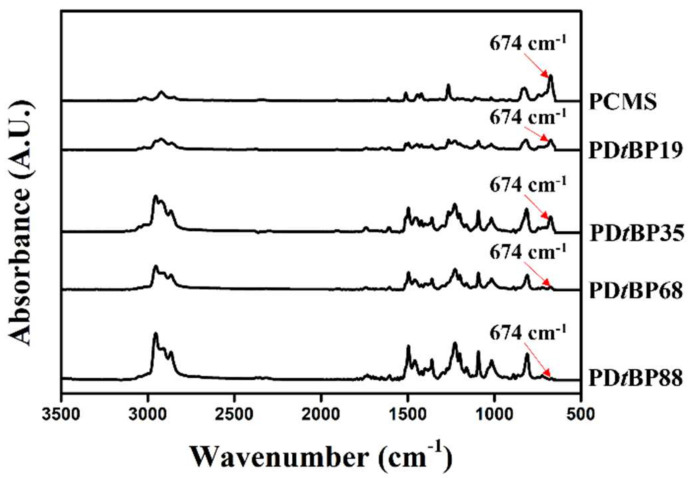
Fourier transform-infrared spectroscopy (FT-IR) spectra of PCMS and PD*t*BP#s.

The synthetic routes to the 2,4-di-*tert*-butylphenoxymethyl-substituted polystyrenes (PD*t*BP88) and copolymers (PD*t*BP68, PD*t*BP35, and PD*t*BP19, where # is the molar content (%) of 2,4-di-*tert*-butylphenoxymethyl side groups) is shown in Figure 1. By varying the amount of 2,4-di-*tert*-butylphenol in reaction, copolymers having different degrees of substitution were obtained as shown in Table 1. The conversion ratios in this substitution reaction of chloromethyl to 1,3-di-*tert*-butylbenzene methyl ether were observed to be lower than the respective feeding ratio of the substituent. It can be explained that a bulky substituent, such as a *tert*-butyl group at the *ortho* position of the phenolic compound causes steric hindrance, thereby reducing the nucleophilicity of the phenolic compound [64]. As shown in Figure 2, the chemical composition of the monomer units in the synthesized polymers can be confirmed through the proton nuclear magnetic resonance (^1^H NMR) spectrum of CMS, PCMS, and PD*t*BP#s and assignment of each peak. The spectrum in Figure 2a indicates the presence of protons in the vinyl group in CMS (δ = 5.2–5.3 (peak b) and 5.7–5.8 (peak c)). The absence of such a peak in Figure 2b means that it was polymerized with PCMS. The spectrum in Figure 2c indicates the presence of protons in the phenyl ring of styrene backbone (*δ* = 1.3–1.6 ppm (peak b)). The proton peaks from the 2,4-di-*tert*-butylphenoxymethyl side chains (*δ* = 1.2–1.3 (peak a), 4.8–5.1 (peak c), and 6.1–7.3 (peak d) indicate the inclusion of 2,4-di-*tert*-butylphenoxymethyl moieties in the polymer. The molar content of 2,4-di-*tert*-butylphenoxymethyl can be calculated by comparing the integral areas of the proton peaks of the 2,4-di-*tert*-butylphenoxymethyl side chain (peak a) and chloromethyl side chains (peak c). ^1^H NMR spectra of PD*t*BP68, PD*t*BP35, and PD*t*BP19 were analyzed in Figure 2d–f through a similar calculation process, respectively. Based on these results, it was concluded that PD*t*BP#s were successfully synthesized. In addition, the chemical structure of each polymer was investigated using ^13^C NMR spectroscopy. Each spectrum is shown in Figures S1–S4. Furthermore, the chemical composition of the monomer units in the synthesized polymers was confirmed by FT-IR spectroscopy, as shown in Figure 3. As the molar contents of 2,4-di-*tert*-butylphenoxymethyl moiety in PD*t*BP# increased, the peak of the characteristic band of stretching vibration mode of chloromethyl in PCMS at 674 cm^−1^ decreased. This result means that the chloromethyl group was changed to a 2,4-di-*tert*-butylphenoxymethyl group through the polymer analogous reactions. These polymers have good solubility in medium-polarity solvents with low boiling temperatures, such as chloroform and tetrahydrofuran, as well as in aprotic polar solvents, for example DMAc, dimethyl sulfoxide (DMSO), and *N*,*N*′-dimethylformamide (DMF).

**Figure 4:**
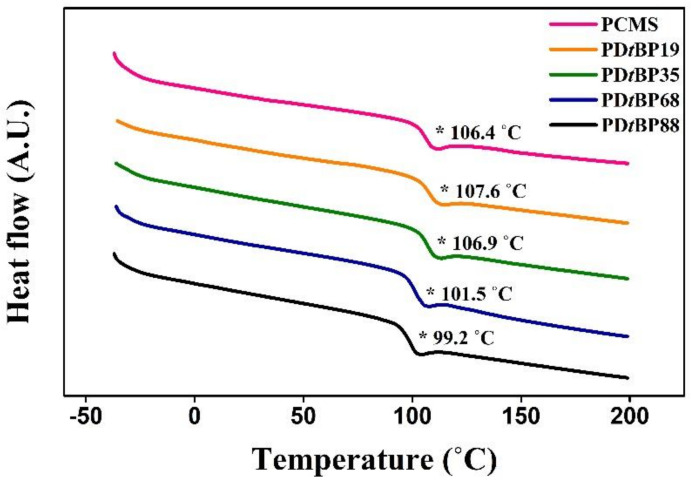
Differential scanning calorimetry (DSC) thermogram of 2,4-di-*tert*-butylphenoxymethyl modified polystyrene (PD*t*BP#). (* means the status of glass transition).

Figure 4 shows the results of investigating the thermal properties of the synthesized polymer using a DSC thermogram obtained from a second heating scan at a rate of 10 °C /min. In addition, the results of the DSC thermograms obtained from the full heating cycles at a rate of 10 °C/min can also be found in Supplementary Material Figures S5–S9. In the DSC thermogram of the copolymer, the melting temperature peak (*T_m_*) was not detected and only the glass transition temperature (*T_g_*) was shown. These results indicate that all samples are amorphous materials. A decrease in *T_g_* of polystyrene derivatives with bulky substituents on the side group has been previously reported [65]; for example, *T_g_* of PD*t*BP68 and PD*t*BP88 is lower than that of polystyrene. Furthermore, as the molar content of 2,4-di-*tert*-butylphenoxymethyl side group increased from 19% to 88%, the *T_g_* value decreased from 107.6 °C for PD*t*BP19 to 99.2 °C for PD*t*BP88. This result can be explained by the decrease in the *T_g_* value because the free volume, which is the space present inside the polymer, increases as the molar content of the bulky side group increases [66]. However, the *T_g_* values increase from PCMS for 106.4 °C to 107.6 °C and 106.9 °C for PD*t*BP19 and PD*t*BP35, respectively. It is known from previous studies that the *T_g_* value of PS derivatives depends on the physicochemical interactions between polymer chains [67]. Therefore, the increase in the *T_g_* values of PD*t*BP19 and PD*t*BP35 rather than that of PCMS is due to the increase in molecular interactions such as π-π interactions and/or van der Waals interactions at the side groups [68,69]. Therefore, the trend of the *T_g_* value is interpreted with the other two analysis points: free volume effect and interaction effect.

### 3.2. LC Orientation Behavior of the LC Cell Fabricated with 2,4-Di-tert-butylphenoxymethyl-Modified Polystyrene Film

**Figure 5:**
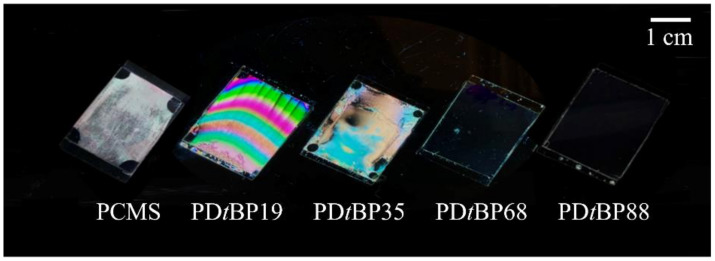
Photograph images of the LC cells made from PD*t*BP# films according to the molar content of 2,4-di-*tert*-butylphenoxymethyl moiety.

A vertically aligned LC device with good display performance can be implemented when the initial orientation of the LCs is uniformly aligned. Figure 5 shows photographic images of LC cells made with PD*t*BP# copolymers to observe the orientation behavior of the LC molecules in PD*t*BP# cells. The LC cells fabricated from PD*t*BP# films with a 2,4-di-*tert*-butylphenoxymethyl side group content of less than 35 mol% (PD*t*BP19 and PD*t*BP35) showed planar LC alignment behavior, while good uniformity of vertical LC alignment behavior was observed for LC cells fabricated with the polymer films with a 2,4-di-*tert*-butylphenomethyl side group content of at least 68 mol% (PD*t*BP68 and PD*t*BP88) in the entire area image area. All of the PD*t*BP88 films could induce stable vertical LC aligning behaviors, and the vertical LC alignment was sustained for at least several months. Therefore, it was confirmed that vertical alignment can be observed in LC cells made of PD*t*BP# films with high molar content of side groups.

**Figure 6:**
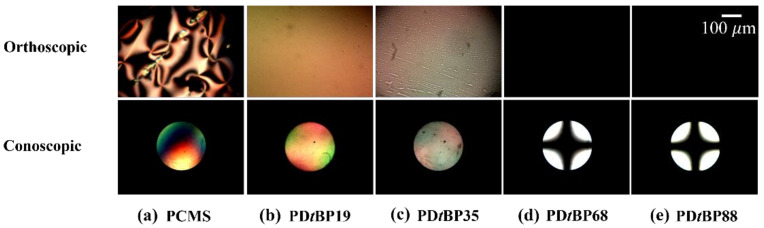
Orthoscopic and conoscopic POM images of the LC cells made from: (**a**) PCMS, (**b**) PD*t*BP19, (**c**) PD*t*BP35, (**d**) PD*t*BP68, and (**e**) PD*t*BP88 films.

The LC aligning behaviors of the LC cells made from PD*t*BP# films were also examined by observing orthoscopic and conoscopic POM images, as shown in Figure 6. When the LC molecules are vertically aligned under cross-polarization, the light is blocked, producing a dark orthoscopic image and a conoscopic image with a Maltese cross pattern. Random planar LC aligning behavior was observed for LC cells made with the poly(4-chloromethylstyrene) (PCMS) film (Figure 6(a)). When the molar content of the 2,4-di-*tert*-butylphenoxymethyl-containing monomeric part in PD*t*BP# was 19 and 35%, the LC cells fabricated using the PD*t*BP# film exhibited random planar LC alignment behavior in the orthoscopic and conoscopic POM images. On the other hand, vertical LC aligning behavior was observed for the LC cells made with the polymeric films PD*t*BP68 and PD*t*BP88, as can be seen in the Maltese cross-pattern of the conoscopic POM images.

### 3.3. Surface Properties of 2,4-Di-tert-butylphenoxymethyl-Modified Polystyrene Films

**Figure 7:**
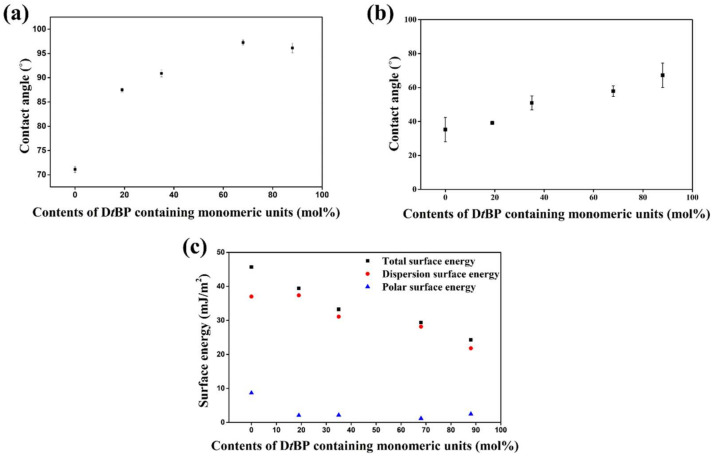
(**a**) Water contact angle, (**b**) diiodomethane contact angle, (**c**) surface energy values of PCMS, PD*t*BP19, PD*t*BP35, PD*t*BP68, and PD*t*BP88 films.

We tried to explain the LC alignment behavior on the PD*t*BP# films using surface characterization techniques. It is known that the high pretilt angles of LCs that produce vertical aligning properties are well correlated with low surface energy values on the alignment film and/or steric repulsion between LCs and the alignment surfaces [70]. Figure 7 and Table 2 indicate the surface energy values of the PCMS, PD*t*BP19, PD*t*BP35, PD*t*BP68, and PD*t*BP88 films obtained based on static contact angles of distilled water and methylene iodide. The total surface energy of polymer film was calculated using the Owens–Wendt’s equation, which is the sum of the polar and dispersion. We also found that the polymer has a critical surface energy value that provides vertical LC alignment behavior. Vertical LC alignment was observed in the PD*t*BP68 and PD*t*BP88 films. The total surface energy values of these polymers were ≤29.4 mJ/m^2^ such as PD*t*BP68 (29.4 mJ/m^2^) PD*t*BP88 (24.3 mJ/m^2^), whereas the PD*t*BP19 and PD*t*BP35, which have total surface energy values ≥33.3 mJ/m^2^, showed no vertical alignment behavior. Therefore, it can be concluded that the vertical aligning capability of PD*t*BP68 and PD*t*BP88 was due to enhanced steric repulsion between LCs and polymeric surfaces caused by introducing bulky and nonpolar 2,4-di-*tert*-butylphenoxymethyl moieties into the side chain of polystyrene, and due to the low polar surface energy (≤29.4 mJ/m^2^) originating from the peculiar molecular structure of the nonpolar carbon containing groups.

### 3.4. Reliability and Optical Performance of the LC Cells Fabricated with 2,4-Di-tert-butylphenoxymethyl-Modified Polystyrene Films

**Figure 8:**
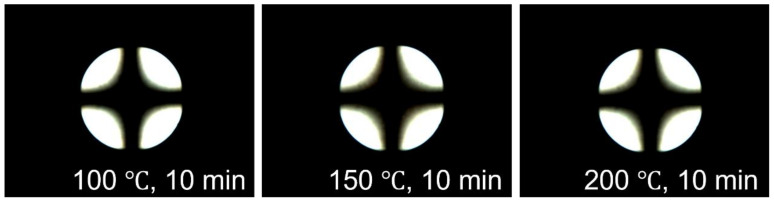
Concoscopic POM images of the LC cells made PD*t*BP88 films, after thermal treatment at 100, 150, and 200 °C for 10 min, respectively.

The reliability of LC cells made of polymer films was investigated by testing the stability of LC alignment in extreme environments such as high temperatures. The thermal stability of the LC cells made from the PD*t*BP88 film was confirmed in the POM image after uniform heating at 100, 150, and 200 °C for 10 min, respectively. As shown in Figure 8, no difference in the pretilt angle on the PD*t*BP88 film with vertical LC alignment ability at 100, 150, and 200 °C was observed through the Maltese cross-pattern in POM images. This indicates that even at a high temperature of 200 °C, the vertical LC alignment ability of the PD*t*BP88 LC cell was maintained. The total surface energy values of the PD*t*BP88 films obtained on the basis of the static contact angles of distilled water and methylene iodide were measured after uniform heating. Even at a temperature of 200 °C, the total surface energy value of the PD*t*BP88 film was maintained in the range of 24.0–25.0 mJ/m^2^. Therefore, as a natural compound modified polymer, PD*t*BP# can be used as a candidate LC alignment layer for sustainable applications.

## 4. Conclusions

A series of polystyrene derivatives containing the common natural product 2,4-di-*tert*-butylphenoxymethyl (PD*t*BP#) were synthesized in order to study the liquid crystal (LC) alignment properties of these PD*t*BP# films. The LC alignment properties were found to be affected by the molar content of the 2,4-di-*tert*-butylphenoxymethyl. For example, LC cells made from films of the polymers with ≥68 mol% of 2,4-di-*tert*-butylphenoxymethyl units (PD*t*BP68 and PD*t*BP88) showed vertical LC alignment. However, LC cells made from PD*t*BP# films with 35 mol% or less of 2,4-di-*tert*-butylphenoxymethyl exhibited random planar LC alignment behavior. In addition, LC cells made from the PD*t*BP68 and PD*t*BP88 films maintained their alignment ability and electro-optical properties for at least several months. The vertical LC alignment was ascribed to steric repulsion between the LC molecules and the polymer surface owing to the incorporation of a non-polar and bulky 2,4-di-*tert*-butylphenoxymethyl moiety into the side chain. In addition, the vertical LC alignment of the PD*t*BP# film is associated with the polymer surface with low total surface energy values (≤29.4 mJ/m^2^). This provides a concept for zero-waste LC alignment layer designs based on natural source-containing polymer films.

## Figures and Tables

**Table 1:** Reaction conditions and results for the synthesis of PD*t*BP.

Polymer Designation	Feed Ratio of 2,4-Di-*tert*-butylphenol (mol%)	Degree of Substitution (mol%)	*T_g_* (°C)
PCMS	0	0	106.4
PD*t*BP19	40	19	107.6
PD*t*BP35	60	35	106.9
PD*t*BP68	80	68	101.5
PD*t*BP88	100	88	99.2

**Table 2:** Surface energy values and LC alignment properties.

Polymer Designation	Contact Angle (°) *^a^*		Surface Energy (mJ/m^2^) *^b^*			Vertical LC Aligning Ability
	Water	Diiodo Methane	Polar	Dispersion	Total	
PCMS	71.1	35.2	8.7	37.0	45.7	No
PD*t*BP19	87.5	39.2	2.1	37.4	39.5	No
PD*t*BP35	90.9	50.9	2.2	31.1	33.3	No
PD*t*BP68	97.3	57.9	1.2	28.2	29.4	Yes
PD*t*BP88	96.1	67.2	2.5	21.8	24.3	Yes

*^a^* Measured from static contact angle. *^b^* Calculated from the Owens–Wendt’s equation.

## Data Availability

The data presented in this study are available on request from the corresponding author.

## Supplementary Materials

The following supporting information can be downloaded at: https://www.mdpi.com/article/10.3390/polym14071302/s1, Figure S1. ^13^C nuclear magnetic resonance (^13^C NMR) spectrum of PCMS; Figure S2. ^13^C nuclear magnetic resonance (^13^C NMR) spectrum of PD*t*BP88; Figure S3. ^13^C nuclear magnetic resonance (^13^C NMR) spectrum of PD*t*BP68; Figure S4. ^13^C nuclear magnetic resonance (^13^C NMR) spectrum of PD*t*BP19; Figure S5. Differential scanning calorimetry (DSC) thermogram of PCMS during the full heating cycles; Figure S6. Differential scanning calorimetry (DSC) thermogram of PD*t*BP88 during the full heating cycles; Figure S7. Differential scanning calorimetry (DSC) thermogram of PD*t*BP68 during the full heating cycles; Figure S8. Differential scanning calorimetry (DSC) thermogram of PD*t*BP35 during the full heating cycles; Figure S9. Differential scanning calorimetry (DSC) thermogram of PD*t*BP19 during the full.

## Abbreviations

The following abbreviations are used in this manuscript:

CMS	4-Chloromethylstyrene
5CB	4′-Pentyl-4-biphenylcarbonitrile
DSC	Differential scanning calorimetry
^1^H NMR	^1^H nuclear magnetic resonance
LC	Liquid crystal
LCDs	Liquid crystal displays
PI	Polyimide
PS	Polystyrene
PCMS	Poly(4-chloromethylstyrene)
PD*t*BP	2,4-Di-*tert*-butylphenoxymethyl-substituted polystyrene
POM	Polarized optical microscopy
