# Supplementary material for: Vertical Alignment of Liquid Crystal on Sustainable 2,4-Di-tert-butylphenoxymethyl-Substituted Polystyrene Films
Source: Polymers (Basel). 2022 Mar 23;14(7):1302. doi: 10.3390/polym14071302 (PMC9002882; doi:10.3390/polym14071302)
Supplement: Supplementary file 1 [file polymers-14-01302-s001.zip › polymers-1628540-supplementary.pdf]

polymers

# Supplementary Materials: Vertical Alignment of Liquid Crystal on Sustainable 2,4-Di-*tert*-butylphenoxymethyl-substituted Polystyrene Films

**DaEun Yang, Chowon Jin and Hyo Kang**

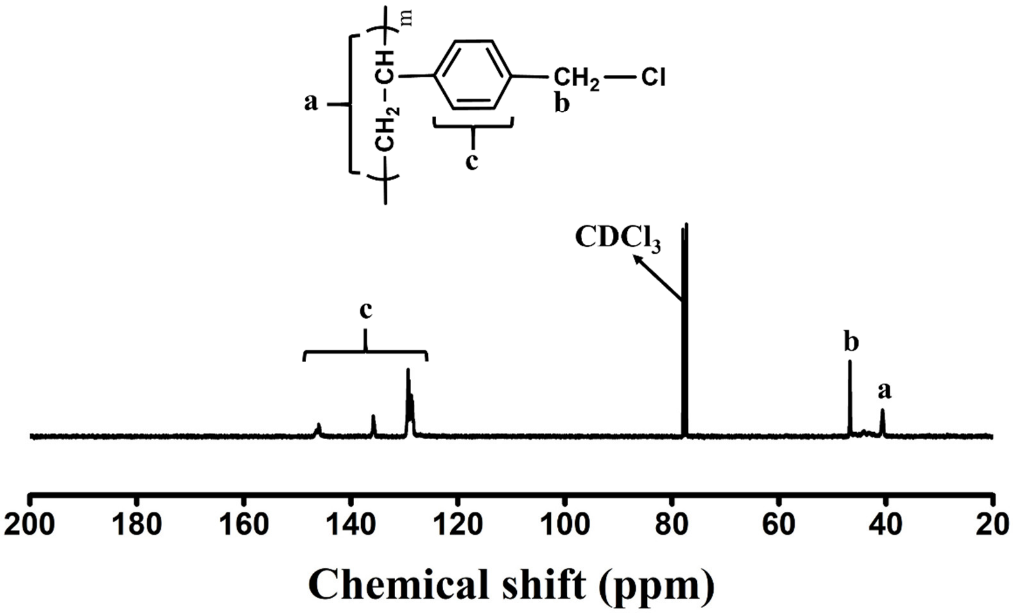


**Figure S1.** $^{13}$C nuclear magnetic resonance ($^{13}$C NMR) spectrum of PCMS.

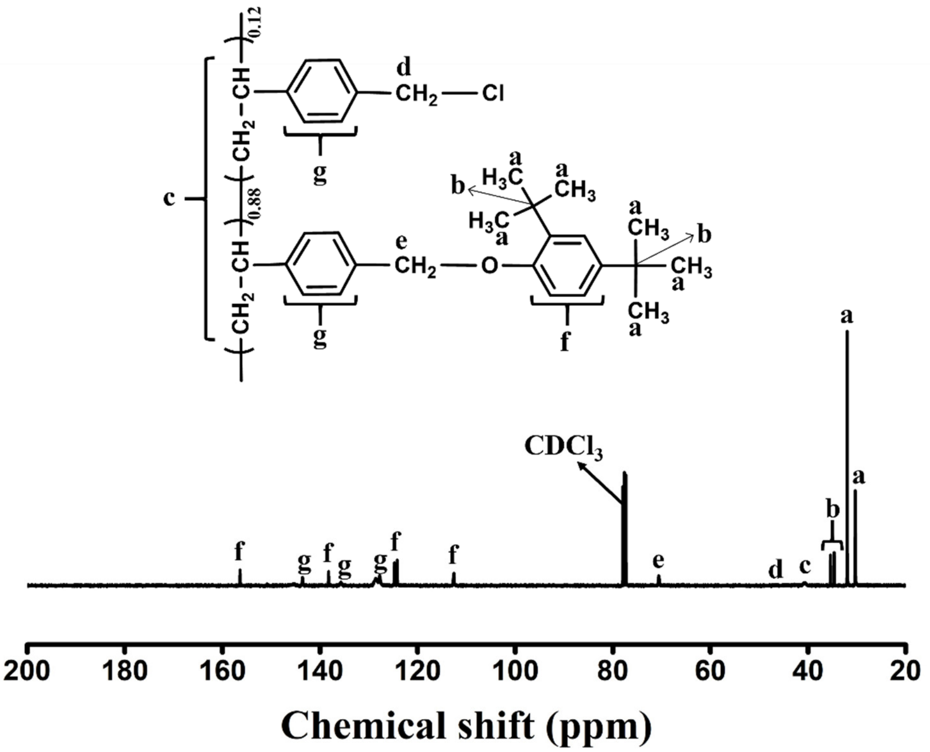


**Figure S2.** $^{13}C$ nuclear magnetic resonance ($^{13}C$ NMR) spectrum of PD*t*BP88.

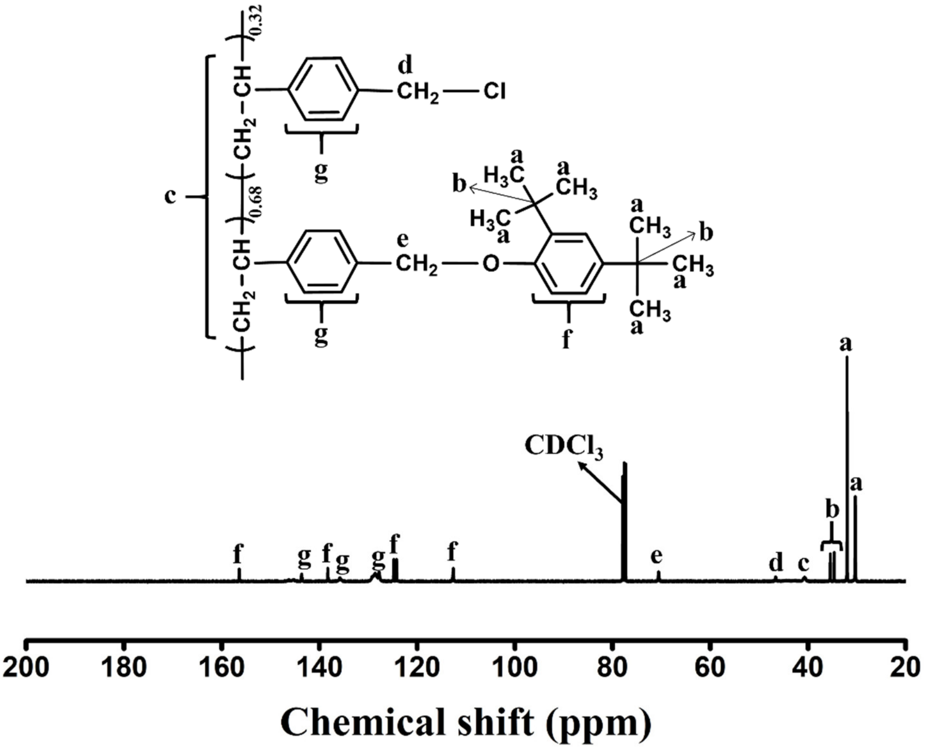


**Figure S3.** $^{13}C$ nuclear magnetic resonance ($^{13}C$ NMR) spectrum of PD*t*BP68.

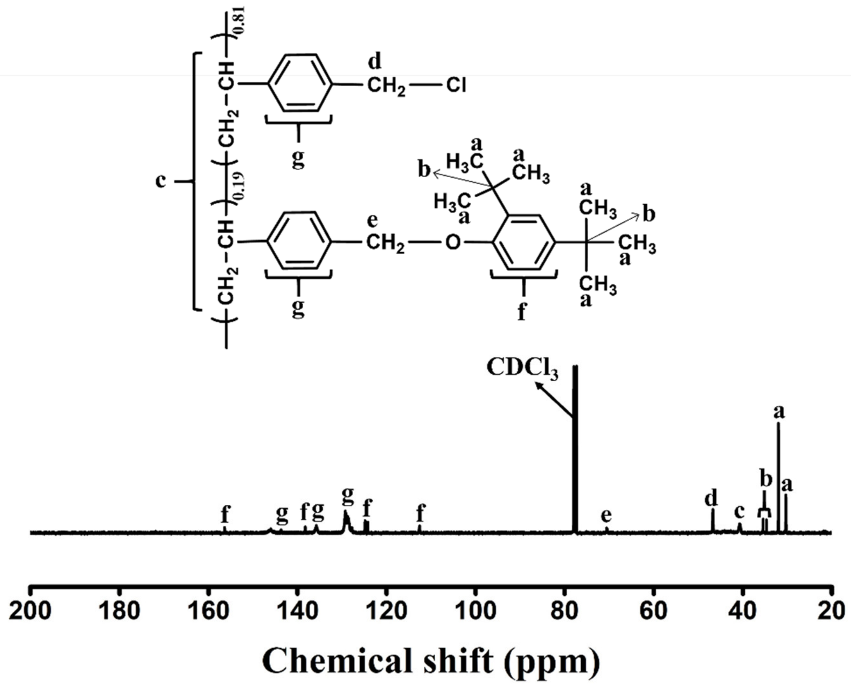


**Figure S4.** $^{13}$C nuclear magnetic resonance ($^{13}$C NMR) spectrum of PD*t*BP19.

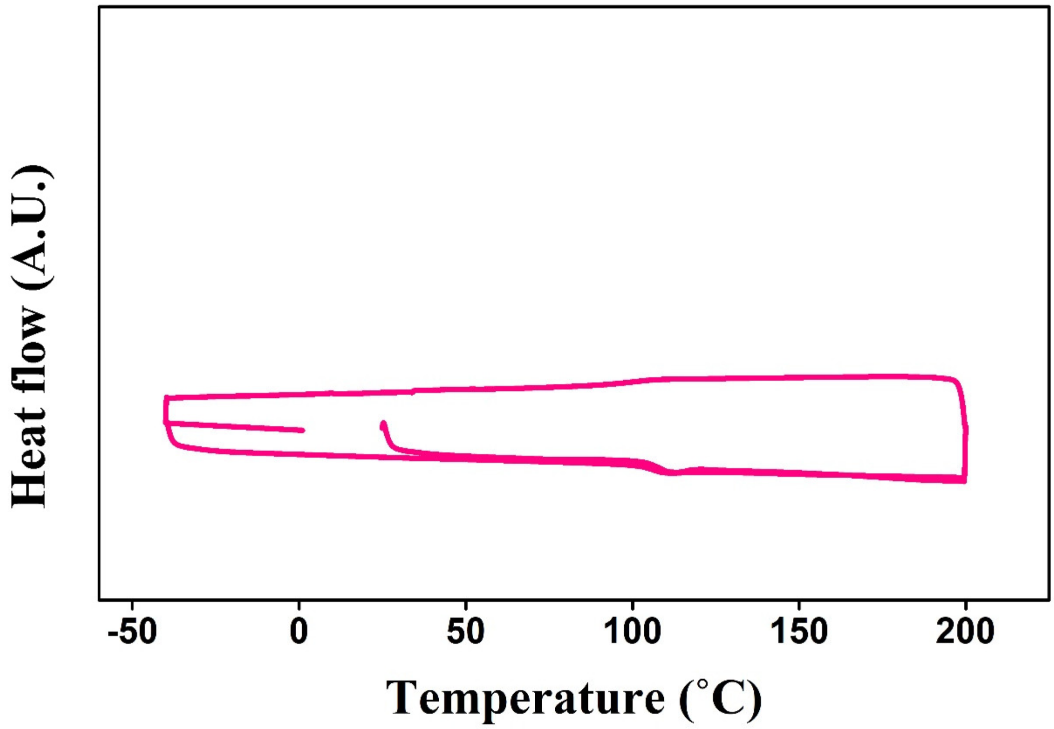


**Figure S5.** Differential scanning calorimetry (DSC) thermogram of PCMS during the full heating cycles.

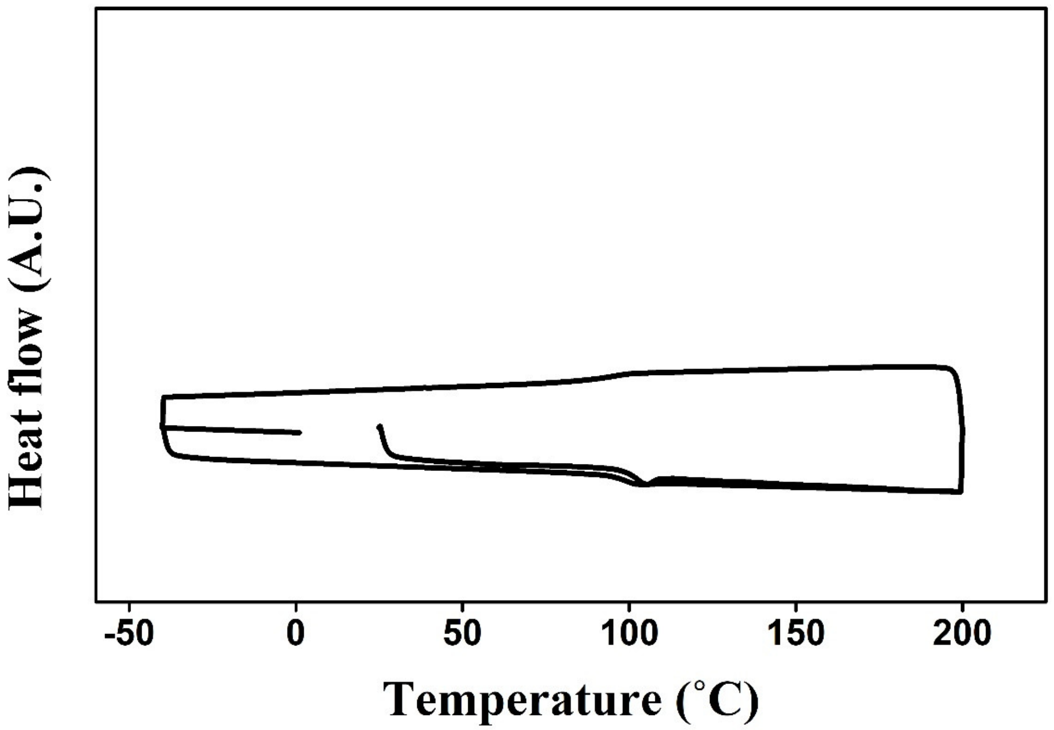


**Figure S6.** Differential scanning calorimetry (DSC) thermogram of PD*t*BP88 during the full heating cycles.

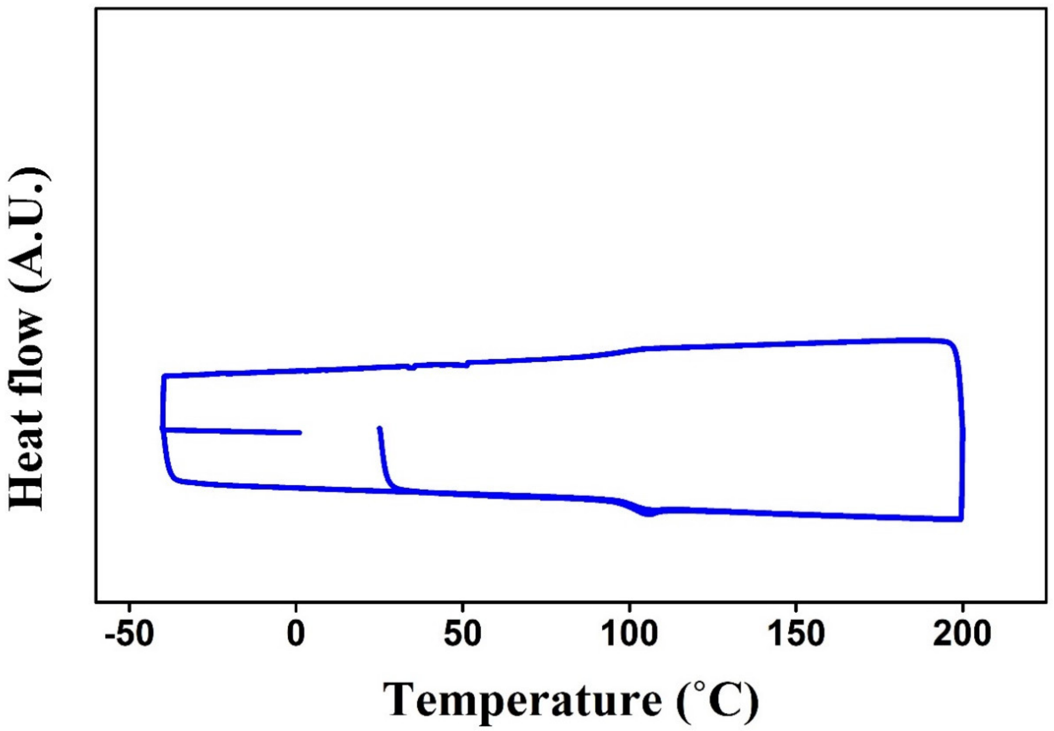


**Figure S7.** Differential scanning calorimetry (DSC) thermogram of PD*t*BP68 during the full heating cycles.

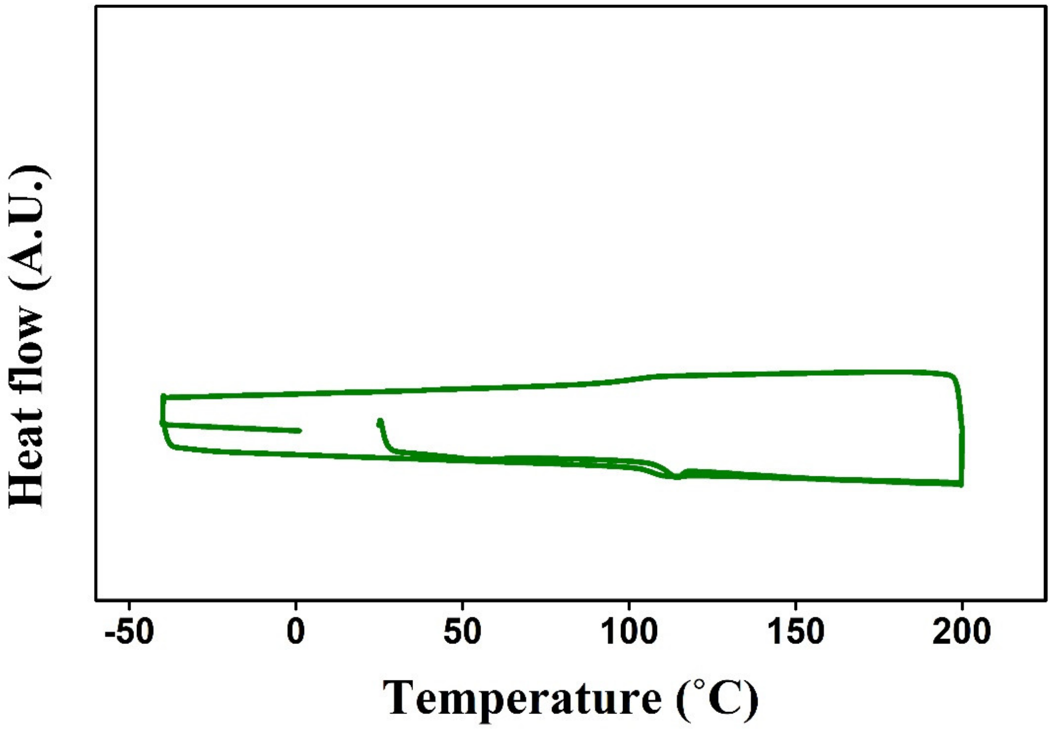


**Figure S8.** Differential scanning calorimetry (DSC) thermogram of PD*t*BP35 during the full heating cycles.

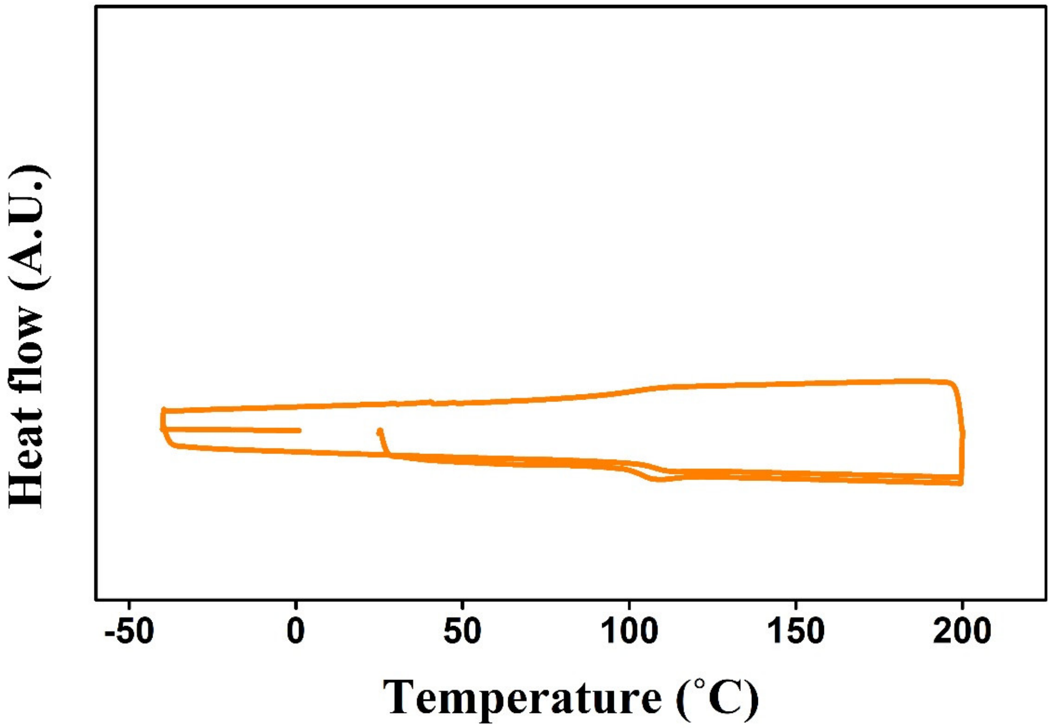


**Figure S9.** Differential scanning calorimetry (DSC) thermogram of PD*t*BP19 during the full heating cycles.
